# Why Creativity Matters to Aging and Health

**DOI:** 10.1093/geroni/igaa057.2208

**Published:** 2020-12-16

**Authors:** Gay Hanna, Pamela Saunders, Marie Bernard

## Abstract

As GSA turns 75, it is an appropriate time to review the history of the creative aging movement. This symposium explores the research, policy and practice of creative aging - past and present, starting in the 1970’s through the efforts of pioneering leaders in the aging, humanities and arts in conjunction with growing support from the newly established National Endowment for the Arts and related aging and health service systems. The foundational research by Gene Cohen, MD PHD and others at the turn of the 21st Century will be described in terms of its building the science to utilize the humanities and arts to scaffold policy and practices that promote the potential of aging through creative expression rather than the pervasive view of aging as a time of loss. Moving towards strength-based approaches to further the development of overall health including brain reserve, physical fitness and social networks, creative aging collaborations will be highlighted as the future of this initiative. Case studies of joint research projects between state departments of both aging and arts in partnership with Universities will demonstration contemporary practices to address major aging issues around isolation, loneliness and caring for the care giver.

